# PPAR*α* Is Required for PPAR*δ* Action in Regulation of Body Weight and Hepatic Steatosis in Mice

**DOI:** 10.1155/2015/927057

**Published:** 2015-10-29

**Authors:** Wojciech G. Garbacz, Jeffrey T. J. Huang, Larry G. Higgins, Walter Wahli, Colin N. A. Palmer

**Affiliations:** ^1^Medical Research Institute, University of Dundee, Ninewells Hospital and Medical School, Dundee DD1 9SY, UK; ^2^Center for Integrative Genomics, University of Lausanne, Le Genopode, 1015 Lausanne, Switzerland; ^3^Lee Kong Chian School of Medicine, Nanyang Technological University, Singapore 308232; ^4^INRA ToxAlim, UMR 1331, Chemin de Tournefeuille, 31300 Toulouse Cedex, France

## Abstract

Peroxisome proliferator activated receptors alpha (PPAR*α*) and delta (PPAR*δ*) belong to the nuclear receptor superfamily. PPAR*α* is a target of well established lipid-lowering drugs. PPAR*δ* (also known as PPAR*β*/*δ*) has been investigated as a promising antidiabetic drug target; however, the evidence in the literature on PPAR*δ* effect on hepatic lipid metabolism is inconsistent. Mice conditionally expressing human PPAR*δ* demonstrated pronounced weight loss and promoted hepatic steatosis when treated with GW501516 (PPAR*δ*-agonist) when compared to wild type mice. This effect was completely absent in mice with either a dominant negative form of PPAR*δ* or deletion of the DNA binding domain of PPAR*δ*. This confirmed the absolute requirement for PPAR*δ* in the physiological actions of GW501516 and confirmed the potential utility against the human form of this receptor. Surprisingly the genetic deletion of PPAR*α* also abrogated the effect of GW501516 in terms of both weight loss and hepatic lipid accumulation. Also the levels of the PPAR*α* endogenous agonist 16:0/18:1-GPC were shown to be modulated by PPAR*δ* in wild type mice. Our results show that both PPAR*δ* and PPAR*α* receptors are essential for GW501516-driven adipose tissue reduction and subsequently hepatic steatosis, with PPAR*α* working downstream of PPAR*δ*.

## 1. Introduction

The rise in number of overweight and obese people worldwide is a major health concern, due to the accompanying metabolic dysfunction and increased hazard for a wide range of fatal illnesses [1]. One of the promising but commercially yet unexplored antiobesity and antidiabetic agents is peroxisome proliferator activated receptor delta (PPAR*δ*) agonists [2, 3].

PPAR*δ* belongs to the superfamily of nuclear receptors. PPARs are transcription factors activated by various fatty acids and their derivatives [4]. All PPARs bind to the peroxisome proliferator response element (PPRE) on the DNA related to the sequence AAAGTAGGTCANAGGTCA [5]. Binding of PPARs to DNA requires that they form heterodimers with retinoid X receptors. In the absence of the ligand, PPAR-RXR heterodimers are actively repressed by recruitment of corepressors and deacetylation of histones and chromatin modifying factors [6]. Three isotypes of PPAR exist. PPAR*α* was the first member of this group of receptors to be characterized as the receptor responsible for chemically induced rodent-specific hepatomegaly and hepatocarcinoma [7–9]. This receptor is also the target for the fibrate family of lipid-lowering drugs. PPAR*γ*, in contrast, is expressed predominantly in adipose tissue and regulates adipogenesis and insulin sensitivity. Drugs from the class of thiazolidinediones insulin sensitizers are activators of PPAR*γ* [10]. PPAR*δ* (also known as PPAR*β*/*δ*) is the remaining member of the subfamily [4]. It is ubiquitously expressed and it has been shown that PPAR*δ* agonism promotes fatty acid oxidation and utilization in both adipose tissue and skeletal muscle [11]. PPAR*δ* selective agonists improve plasma lipid profile [12] and may inhibit atherosclerosis progression [13, 14]. However, concerns have been raised about the possibility that PPAR*δ* agonists may promote some type of cancer [15]. Another concern regarding toxicological issues with PPAR*δ* agonists relates to the role of PPAR*δ* in hepatic lipid metabolism. Evidence is accumulating that PPAR*δ* can stimulate temporary [16] or more severe [17] fatty acid accumulation in mouse and potentially human liver. This may be a problem as individuals with metabolic syndrome or type 2 diabetes are greatly susceptible to fatty liver steatosis. However, other reports like from Qin and colleagues [18] contradict this hypothesis. Therefore, we sought to test the hypothesis that PPAR*δ* agonism may carry a risk of promoting hepatic fatty accumulation similar to Nonalcoholic Fatty Liver Disease (NAFLD). In order to investigate the role of PPAR*δ* signalling in the regulation of hepatic triglyceride accumulation, we performed* in vivo* experiments using humanised, transgenic animals and different diets and chemical treatments.

## 2. Experimental Procedures

### 2.1. Reagents

PPAR*δ*, {2-methyl-4-{{4-methyl-2-[4-(trifluoromethyl)phenyl]-5-thiazolyl}methylthio}phenoxy}acetic acid (GW501516), was synthesised by AF ChemPharm Ltd., Sheffield, UK. Other chemicals used for this study were purchased from Sigma/Aldrich, Gillingham, Dorset, UK.

### 2.2. Animals

Nontransgenic (non-tg) C57BL/6 mice were obtained from Harlan Laboratories (Harlan, UK). PPAR*α* knockout mice (C57BL/6.129S4-*Ppara* ^*tm1Gonz*^) were purchased from Jackson Laboratory (Bar Harbor, Maine, USA). PPAR*δ* null animals were obtained from University of Lausanne, Switzerland, from Walter Wahli's Laboratory, and maintained on a C57BL/6 background [19]. Generation of mice (C57BL/6) conditionally expressing human PPAR*δ* (hPPAR*δ*) and dominant negative derivative of human PPAR*δ* (hPPAR*δ*ΔAF2) was described elsewhere [20, 21]. In short: transgene expression is controlled by the* Cyp1a1* promoter system allowing for conditional expression of the transgene [22]. The expression of the transgene of choice is dependent on the activation of the mouse endogenous aryl hydrocarbon receptor (AhR). Activation is achieved by dietary administration of the AhR agonist indole-3-carbinol (I3C), 0.25% (w/w). Basal transcription from the* Cyp1a1* promoter in the absence of AhR agonist is very low, allowing for tight control of transgene expression. All mice were fed* ad libitum* and were kept under 12-hour light/dark cycles in humidity and temperature-controlled environment. All procedures were done in accordance with regulations contained in the Animals and Scientific Procedures Act (1996) of the United Kingdom and with the approval of the University of Dundee ethical committee. The animals were fed normal chow (standard RM1 laboratory animal feed, SDS Ltd., Wickham, UK) or chow supplemented with 0.0025% GW501516 (w/w) (4 mg/kg/day). At the termination of each experiment, animals were fasted overnight and sacrificed using increasing concentration of CO_2_. Blood was removed using cardiac puncture, followed by organ removal (liver, muscle (quadriceps), and adipose tissue (visceral fat pad)). Collected tissues were snap-frozen in liquid nitrogen and stored in −80°C until further processing. In all experiments both genders were used in balanced distributions with 5 animals/group, except PPAR*α*-KO experiment, where 4 animals/group were used. All mice used were 10 weeks old at the start of each experiment. No gender differences in the current phenotypes were observed.

### 2.3. Blood and Liver Lipids Measurements

Total lipids from liver were extracted using Folch method [23]. Analysis of plasma and liver lipids was performed using RX Daytona clinical analyzer (Randox, UK) in accordance with manufacturer's instructions.

### 2.4. Body Fat Measurement

Magnetic Resonance Imaging System EchoMRI-4in1 (Houston, Texas, USA) was used to determine body composition in live animals.

### 2.5. Gene Expressions

Total RNA from the liver and muscle was prepared using the RNEasy or RNeasy Mini Fibrous kit (Qiagen) following manufacturer instructions (Qiagen, UK). For the quantitative analysis of genes expression, total RNA was normalized and reverse-transcribed using the High Capacity cDNA Reverse Transcription Kit (Applied Biosystems). Ct values of the genes of interest were normalized to 18s RNA using the delta-delta Ct method. Taqman probes and primers were previously described [13] or were designed using Primer Express 3.0 and were purchased from Applied Biosystems (UK) or from Eurofins MWG Operon (London, UK) or from Sigma/Aldrich, Gillingham, Dorset, UK. Primer and probe sequences are shown in Supplementary Table 1 (in Supplementary Material available online at http://dx.doi.org/10.1155/2015/927057). The ABI Prism 7900 sequence detector (Applied Biosystems) was used to perform RT-PCR reaction and the data was acquired and processed with Sequence Detector 1.6.3 software (Applied Biosystems).

### 2.6. Microarray Analysis

Total RNA was harvested from liver samples (5 animals per group) using the RNeasy kit (Qiagen) according to the manufacturer's instructions, including a DNase digestion step. Total RNA was quantified on a NanoDrop spectrophotometer (ND-8000) and samples were amplified and labelled using Illumina TotalPrep RNA Amplification Kit (Invitrogen, UK). All samples passed quality check using Agilent 2100 Bioanalyser and 1.5 *μ*g/biotin-labelled sample was hybridized with MouseWG-6 v2.0 Expression BeadChips (Illumina, USA). BeadChips (*n* = 60) were scanned using an Illumina BeadArray reader and raw data were acquired using GenomeStudio software. Expression data were normalized in GenomeStudio with background extraction. Microarray data were analyzed using GenomeStudio, MeV v. 4.8.1. and MS Excel. Group means for weight gain (% of initial body mass) and hepatic triglyceride levels at point of 2 weeks were established from data from previous experiments involving non-tg fed control or diet supplemented with GW501516; non-tg, hPPAR*δ*, and hPPAR*δ*ΔAF2 fed control + 0.25% I3C or diet supplemented with 0.25% I3C + 0.0025% GW501516; PPAR*α*-KO and PPAR*δ*-KO fed control or diet supplemented with GW501516 (w/w). Phenotypic group means from experimental 2-week time point were then correlated with hepatic gene expression data obtained from 5-day experiment. Out of 30854 genes (consisting of 45281 probes) available on the microarray chips, 9548 reached intensities higher from background with a mean *P* value over the 60 samples of <0.1. This broad selection was required to not exclude genes that were nonexpressed in specific samples, for example, the nulls, or where expression was only significantly detectable in the induced samples. False discovery rate method (FDR) [24] was used to correct for multiple testing effects. Overall correlation was considered to be significant if correlation *P* value FDR below 0.05 was obtained.

### 2.7. Measurement of Phosphatidylcholine (GPC)

Quantification of 16:0/18:1-GPC was according to a previous report [25] with some modifications. In brief, the analysis was performed on a Thermo Finnigan TSQ Quantum Ultra triple quadrupole mass spectrometer (ThermoFinnigan, San Jose, CA) in conjunction with a Thermo Dionex Ultimate 3000 LC and an electrospray source. The system was tuned using 16:0/18:1-GPC. Five microliters of lipid extracts was injected to a Phenominex C18 column (50 × 2.1 mm) with a 6-minute LC gradient elution from 50 : 50 CH_3_CN/H_2_O (solution A) to 90 : 10 isopropanol/CH_3_CN (solution B). Both mobile phase solutions contained 0.1% formic acid. The lipids species containing phosphocholine were analyzed in a precursor ion scan (positive ion mode, mass range = 740–820) monitoring neutral loss of 184.1, which corresponds to phosphocholine. The relative abundance of *m*/*z* 760 (representing 16:0/18:1-GPC) to *m*/*z* 758 (representing 16:0/18:2-GPC) was calculated.

### 2.8. Histological Analysis of Liver Tissue

To visualise fat deposits in liver tissue sections, Oil Red O staining was used. Frozen sections of formalin fixed liver (5 *μ*m) were stained with ORO and counterstained with Mayer's hematoxylin.

### 2.9. Statistical Analysis

GraphPad Prism 5.0 (Graphpad Software, Inc, CA, USA), MS Excel, and GenomeStudio, MeV v. 4.8.1 were used for statistical analysis. Two-way ANOVA with Bonferroni post tests, Student's *t*-test, and correlation was used to calculate statistical significance. *P* < 0.05 was considered as significant. All error bars are shown as standard error mean.

## 3. Results

### 3.1. PPAR*δ* Agonism in Nontransgenic Mice

**Figure 1:**
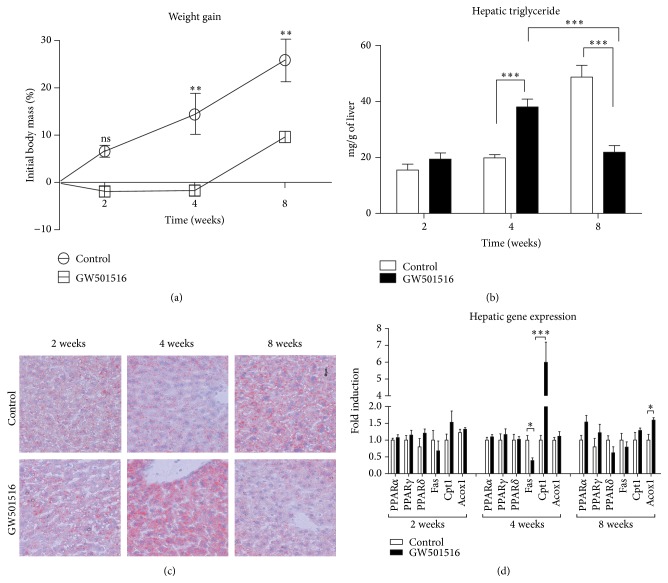
Treatment with GW501516 prevents diet induced weight gain and changes the hepatic TG level in non-tg animals. (a) GW501516 decreased body mass accumulation, expressed as percentage of initial body weight. (b) Following 4 weeks of treatment, TG in the liver increased by 91% (*P* < 0.001) when compared to control animals and after 8 weeks of treatment, hepatic TG levels had returned (decreased by 73%) to levels observed at the 2-week time point in both groups (*P* < 0.001). (c) Liver section stained for fat with Oil Red O. Level of red colour indicates presence of lipid droplets. (d) Chosen hepatic gene expression shows no change in mRNA levels of any member of PPAR family throughout the experiment. Lipogenic gene Fas expression was downregulated (*P* < 0.01) after 4 weeks, while *β*-oxidation markers Cpt1 and Acox1 were upregulated (*P* < 0.001) or remained without change, respectively (4 weeks). *n* = 5 mice/group, two-way ANOVA was used to calculate significance.

In order to test literature-reported hepatic lipid fluctuations in response to PPAR*δ* ligand, nontransgenic mice were fed with diet enriched with GW501516 for 8 weeks along with the control group fed normal chow. Animals were sacrificed at 2, 4, and 8 weeks' time points. PPAR*δ* agonist treatment reduced weight gain in non-tg mice (calculated as a percentage of initial weight). The influence of the PPAR*δ* ligand on weight gain after 4 and 8 weeks of experiment was considered very significant (*P* < 0.01; Figure 1(a)).

In treated animals, plasma level concentration of GW501516 was found to be approximately 1 *μ*mol/L at 2, 4, and 8 weeks' time point (data not shown). The hepatic triglyceride (TG) content in treated animals after 2-week period did not differ significantly between the groups. However, after 4 weeks, the liver TG content in the ligand treated animals increased by 91% (*P* < 0.001) in comparison to control group. At the end of the experiment (8 weeks), the TG in the livers of treated mice were lower by 58% (*P* < 0.001), when compared to control group. When comparing treated groups only, hepatic fat content decreased by 73% (*P* < 0.001) between 4 and 8 weeks (Figure 1(a)).  Figure 1(c) shows pictures of liver sections stained for fat (Oil Red O). Blood lipid profile also revealed that plasma triglyceride levels were found to be statistically lower at every time point in GW501516 treated group, when compared to control animals (data not shown).

Results from gene expression analysis have shown that, in livers of the PPAR*δ* ligand treated animals sacrificed after 4 weeks, there was no upregulation or downregulation of any members of PPAR family (Figure 1(d)). No differences were observed in pattern of hepatic expression of PPARs throughout the whole experiment. Considering the fact that source of the increased liver fat could be due to* de novo* fatty acid synthesis, levels of hepatic Fatty Acid Synthase (Fas) mRNA were measured. Surprisingly, Fas mRNA levels were downregulated in treated animals after 4 weeks by 60% (*P* < 0.01; Figure 1(d)). An impaired *β*-oxidation process could also play role in ectopic fat accumulation. In treated animals hepatic levels of acyl-coenzyme A oxidase 1 (Acox1), the first enzyme of the fatty acid *β*-oxidation pathway, were not different from those found in controls (Figure 1(d)). On the other hand, the expression of carnitine palmitoyltransferase I (Cpt1), encoding an enzyme involved in transport of fatty acids into mitochondria, increased 6-fold in the liver after 4-week treatment in comparison to control group (*P* < 0.001; Figure 1(d)). After 8 weeks, no difference between groups in Cpt1 expression was found.

### 3.2. GW501516 Stimulated Hepatic Lipid Accumulation Is Mediated by a PPAR*δ* Activation-Dependent Mechanism

To test the hypothesis that PPAR*δ* signalling is specifically required for hepatic lipid accumulation in response to GW501516, we used animals conditionally overexpressing human PPAR*δ* (hPPAR*δ*) or conditionally overexpressing dominant negative form of human PPAR*δ* (hPPAR*δ*ΔAF2) along with nontransgenic mice for control.

Mice were divided into control and treatment groups, and all animals were with chow fed diet supplemented with indole-3-carbinol (I3C) (0.25% (w/w)) to induce the transgene in transgenic animals. I3C is a compound naturally occurring in cruciferous plants and when ingested is then converted into polyaromatic indolic compounds, which activate endogenous aryl hydrocarbon receptor (AhR), which is highly expressed in the liver [26]. Treatment group were fed diet containing 0.25% I3C (w/w) and 0.0025% of GW501516. Due to susceptibility of hPPAR*δ* mice to the psoriasis-like skin disease when treated for prolonged periods with GW501516 [20], all groups of animals were treated for 2 weeks only which was previously established as psoriasis phenotype-free.

**Figure 2:**
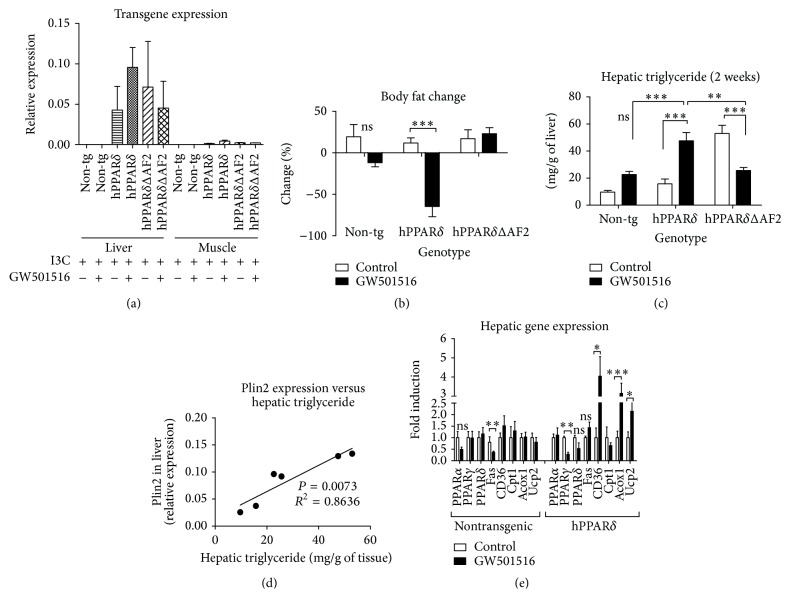
GW501516 stimulated hepatic triglyceride accumulation is mediated through PPAR*δ* action. (a) Transgene expression in liver and muscle in non-tg, hPPAR*δ*, and hPPAR*δ*ΔAF2 animals in 2 weeks. (b) Body fat change determined by Magnetic Resonance Imaging (MRI) in animals fed control diet or diet supplemented with GW501516 in 2 weeks. (c) Liver fat content increased in mice overexpressing hPPAR*δ* and treated with GW501516 in comparison to controls (*P* < 0.001). Mice overexpressing hPPAR*δ*ΔAF2 (treated animals) accumulated less fat in the liver than control animals (*P* < 0.001). (d) mRNA levels of Plin2, a protein marker of TG accumulation and direct PPAR*δ* responsive gene, are significantly correlated with hepatic TG levels. Each point on the graph represents mean value of each group (control and treated) for Plin2 relative expression versus mg/g of hepatic TG. (e) Gene expressions in livers of nontransgenic and hPPAR*δ* mice. Lipogenic master gene PPAR*γ* was downregulated (*P* < 0.01) in GW501516 treated hPPAR*δ* animals, whereas *β*-oxidation enzyme Acox1 was upregulated (*P* < 0.01) (hPPAR*δ*). All genes were normalized to 18s RNA. *n* = 5 mice/group, tested by two-way ANOVA.

Supplementing the diet with I3C (0.25% w/w) resulted in high expression of the transgene mRNA levels in liver (Figure 2(a)). In muscle, however, levels of expression of both transgenes were particularly low when compared to hepatic expression (Figure 2(a)). The transgene is expressed in liver and other organs at extremely low levels in the absence of I3C [21], which confirms that regulated expression of hPPAR*δ* transgene and action of AhR converges in the liver.

Conditional expression of human hPPAR*δ* had a significant effect on weight gain in mice fed diet containing GW501516 as previously described [21], with ligand treated hPPAR*δ* animals having lost 15% of their body weight in 2 weeks, when compared to untreated littermates (*P* < 0.001). Non-tg mice fed diet containing GW501516 and animals conditionally overexpressing hPPAR*δ*ΔAF2 with GW501516 in diet had insignificant differences in body mass or maintained normal weight gain after 2 weeks' time (control versus treated; non-tg and hPPAR*δ*ΔAF2, resp. (data not shown)). Most of the body mass lost was due to fat loss as determined by MRI scanning (Figure 2(b)).

The PPAR*δ* agonist also affected plasma HDL levels. In 2 weeks, the level of HDL in mice overexpressing hPPAR*δ* fed diet containing GW501516 increased by 52% (*P* < 0.001) (in non-tg the increase in HDL was noticeable but not significant), whereas in hPPAR*δ*ΔAF2 animals no significant differences between groups were observed (data not shown).

Dissection of the animals revealed a markedly pale colour of the livers in the animals overexpressing hPPAR*δ* fed diet enriched GW501516 and in the hPPAR*δ*ΔAF2 animals on control diet. Further direct lipid measurement confirmed increase in hepatic TG in hPPAR*δ* mice treated with GW501516 by 202%, when compared to untreated controls (*P* < 0.001, two-way ANOVA) (Figure 2(c)). hPPAR*δ*ΔAF2 animals fed control diet also had elevated liver TG comparable to the hPPAR*δ* mice with the ligand in the diet. However, the group of hPPAR*δ*ΔAF2 animals treated with GW501516 had lower level of hepatic TG by 50%, compared to their untreated controls (*P* < 0.001) (Figure 2(c)). The GW501516 presence in hPPAR*δ*ΔAF2 mice appears to restore repressor function of this receptor, which is consistent with the reverse agonism that we have seen in other experiments [21].

Gene expression analysis revealed that, in liver after 2 weeks, levels of mRNA of adipophilin (Adipose Differentiation-Related Protein, ADRP, also known as perilipin 2 or Plin2) were highly correlated (*P* = 0.0073; *R* ^2^ = 0.863) with the levels of hepatic TG in both control and treated groups and across the genotypes of animals (Figure 2(d)). Plin2 encodes protein that coats lipid droplets and it is a direct PPAR*δ* target gene but also reported to be responsive to PPAR*α* [27] and therefore can serve as a marker of both PPAR*δ* activation and TG accumulation.

No significant difference between groups in Fas mRNA levels was found in mice conditionally overexpressing human PPAR*δ* (Figure 2(e)). Additionally, hepatic transcript levels of PPAR*γ*, a master of lipogenic genes, were lower in agonist treated mice by 72% (*P* < 0.01) (Figure 2(e)). On the other hand, mRNA for CD36 fatty acid transporter was increased in treated animals. The mRNA levels of *β*-oxidation marker, Acox1, were increased by 214% in GW501516 treated animals (hPPAR*δ*) when compared to control group (*P* < 0.01) and uncoupling protein 2 (Ucp2) mRNA was also positively changed in treated mice (Figure 2(e)).

### 3.3. PPAR*α* Downstream Signalling Is Essential for PPAR*δ* Agonist-Induced Weight Loss and Liver Steatosis

**Figure 3:**
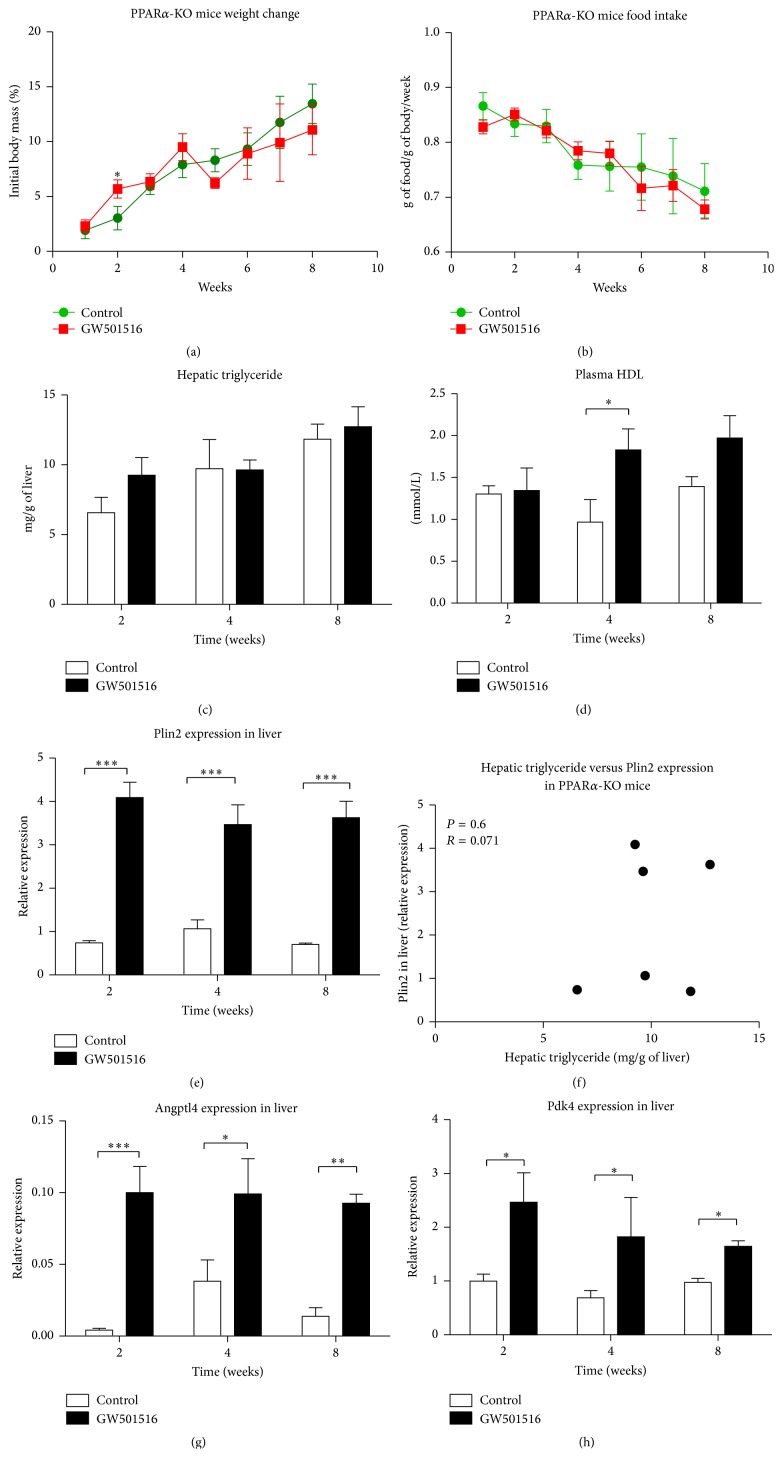
PPAR*α* receptor is essential for GW501516 induced weight loss and hepatic steatosis. (a) GW501516 dependent weight loss is not evident in PPAR*α*-KO mice. (b) Both groups matched in their weekly food intake. (c) No significant difference was found in hepatic TG between PPAR*α*-KO mice fed control or a diet supplemented with GW501516. (d) Nonfasted PPAR-KO mice had still detectable rise in plasma HDL when fed diet containing GW501516. (e) GW501516 activates PPAR*δ* in PPAR*α*-KO mice. mRNA levels of Plin2, a marker of PPAR*δ* activation, were upregulated in PPAR*α*-KO mice following feeding with a diet supplemented with GW501516. (f) mRNA expression level of hepatic Plin2 was not correlated with the level of liver TG. Each point on the graph represents mean value of each group for Plin2 relative expression and for mg/g of hepatic TG. Two more PPAR*δ* target genes changed their mRNA expression level after treatment with GW501516 in PPAR*α*-KO animals. Angptl4 (g) and Pdk4 (h) were upregulated in liver. Significance is indicated (^*∗*^ *P* ≤ 0.05; ^*∗∗*^ *P* ≤ 0.01; ^*∗∗∗*^ *P* ≤ 0.001), *n* = 4 mice/group.

The long-term clearance of PPAR*δ*-dependent liver fat accumulation described in experiment with non-tg animals was evident and occurs despite stable GW501516 plasma levels at every time point (8-week experiment, Figure 1(b)). We assumed that PPAR*α* activation and signalling could be responsible for removal of the liver fat accumulated by PPAR*δ* agonism. To investigate whether this hypothesis is true, PPAR*α* receptor null mice (PPAR*α*-KO) were used. PPAR*α*-KO mice are known to be susceptible to fasting induced hepatic steatosis [28, 29]; therefore before the sacrifice, mice were not fasted. Animals were divided into 2 groups, one control fed normal chow and the treatment group fed diet supplemented with 0.0025% GW501516 (w/w). Mice were sacrificed at 2, 4, and 8 weeks from beginning of the experiment. Surprisingly, PPAR*α*-KO mice fed GW501516 had lost no weight during the whole experiment, when compared to control animals fed normal chow (Figure 3(a)). There was no difference in food intake between both groups throughout the whole length of experiment (Figure 3(b)). Lipid measurements revealed no liver steatosis in PPAR*α*-KO mice treated with PPAR*δ* ligand (Figure 3(c)). Differences in hepatic TG content between groups were not significant at any time point of experiment. However, despite the lack of functional PPAR*α*, the rise in plasma HDL levels upon GW501516 treatment was still detectable, with only 4 weeks' time measurement difference being statistically significant (*P* < 0.05) (Figure 3(d)).

Although fatty liver phenotype and PPAR*δ*-dependent weight loss were completely abolished in PPAR*α*-KO mice fed PPAR*δ* agonist, gene expression in that group showed that many direct PPAR*δ* target genes were still upregulated. Liver Plin2 mRNA levels were significantly higher at every time point (3–5.5-fold) (*P* < 0.001) (Figure 3(e)). However, no correlation was found between Plin2 and hepatic TG (Figure 3(f)). The angiopoietin-related protein 4 (Angptl4) is involved in lipid metabolism and is the target of PPAR*δ*. Hepatic Angptl4 mRNA levels were also significantly elevated in treatment groups (Figure 3(g)). In addition, hepatic mRNA levels of pyruvate dehydrogenase kinase isozyme 4 (Pdk4), another PPAR*δ* and PPAR*α* target gene [30], were increased. Pdk4 phosphorylates pyruvate dehydrogenase complex, thus inhibiting carbohydrate metabolism (Figure 3(h)).

**Figure 4:**
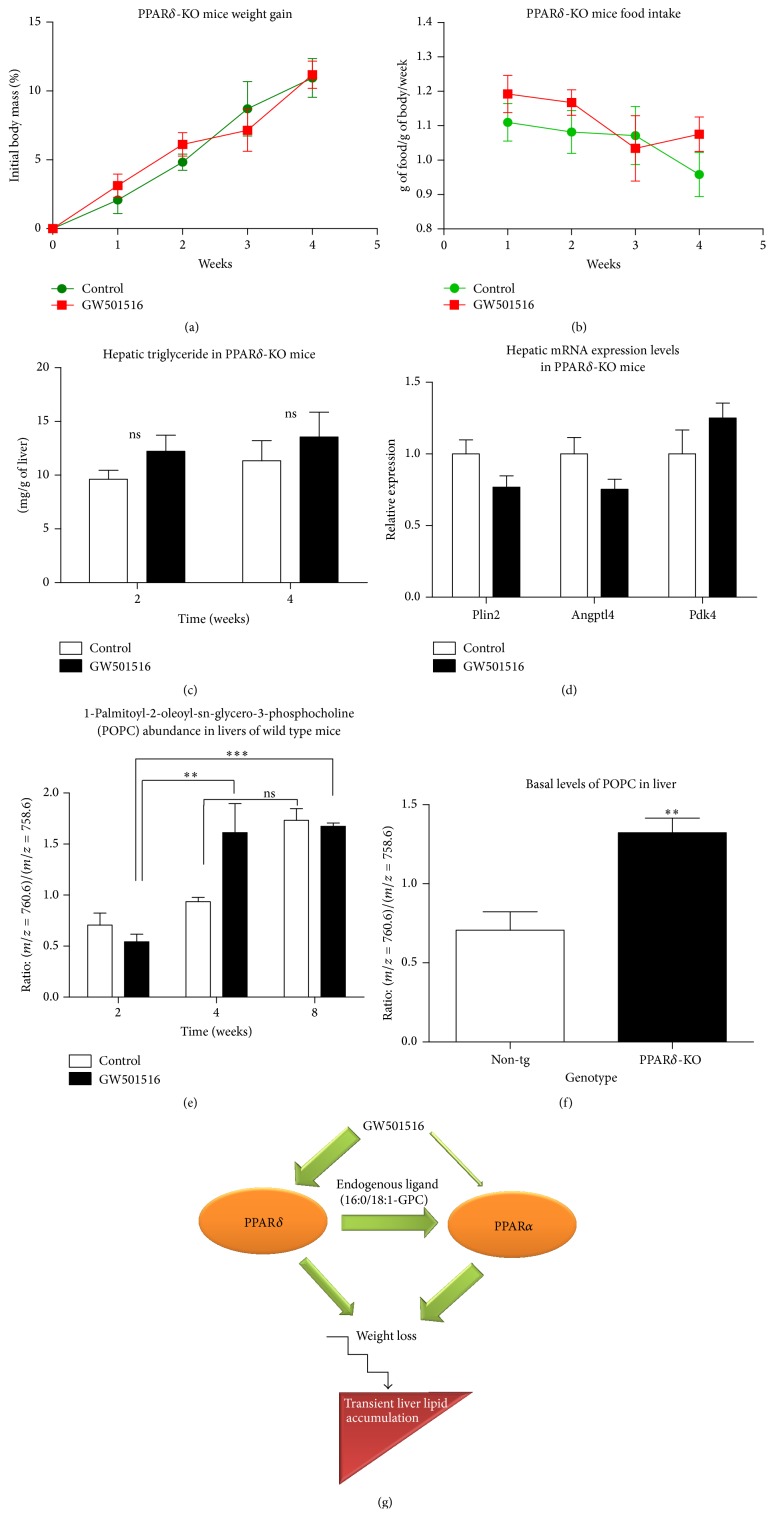
Lack of GW501516 effect in PPAR*δ*-KO mice and hepatic levels of endogenous ligand for PPAR*α*. GW501516 treatment had no significant overall influence on weight gain (a), food intake (b), or hepatic lipids (c) in PPAR*δ*-KO mice. (d) Hepatic mRNA expression levels of PPAR*δ* downstream target genes such as Plin2, Angptl4, and Pdk4 were not changed by GW501516 treatment in PPAR*δ*-KO animals. (e) 1-Palmitoyl-2-oleoyl-sn-glycero-3-phosphocholine (POPC) (endogenous PPAR*α* ligand) increase over time upon GW501516 treatment in non-tg mice. (f) Relative levels of POPC in livers of PPAR*δ*-KO animals are higher when compared to non-tg mice. (g) Phenotypic effects of PPAR*δ* agonist GW501516 are entirely dependent on downstream PPAR*α* signalling. Genetic ablation of either of these two receptors results in resistance to GW501516-promoted weight loss and liver lipid accumulation. PPAR*α* appears to be downstream of PPAR*δ* potentially activated by endogenous ligand POPC levels determined by PPAR*δ*. Significance is indicated (^*∗*^ *P* ≤ 0.05; ^*∗∗*^ *P* ≤ 0.01; ^*∗∗∗*^ *P* ≤ 0.001; *t* test), *n* = 5 mice/group.

Similar experiment was conducted using PPAR*δ* knockout animals. In 4 weeks' time C57BL/6 PPAR*δ*-KO mice were fed normal chow or diet enriched with 0.0025% GW501516 (w/w). PPAR*δ* agonist treatment did not cause weight loss when compared to control group (Figure 4(a)) and did not alter food intake (Figure 4(b)). No differences were found between control and ligand treated groups in hepatic lipid content (Figure 4(c)) or in mRNA gene expression levels of PPAR*δ* downstream target genes such as Plin2, Angptl4, and Pdk4 (Figure 4(d)).

The hypothesis that PPAR*δ* activation leads to steady build-up of endogenous PPAR*α* ligand [31, 32], thus providing a role for PPAR*α* downstream of PPAR*δ*, was tested. The proposed PPAR*α* endogenous activator 1-palmitoyl-2-oleoyl-sn-glycero-3-phosphocholine (POPC) was detected in whole hepatocyte lipid extracts using LC-MS analysis. The time was the key factor in increasing levels of POPC in livers of non-tg animals fed diet enriched with GW501516 throughout the length of the study, where the difference within the treatment groups between 2 and 4 and 8 weeks was 3-fold in favour of the latter ones (*P* = 0.0065 and *P* < 0.001, resp.) (Figure 4(e)). Basal hepatic levels of POPC from 2 weeks' time point in PPAR*δ*-KO animals were also significantly higher than in non-tg mice (Figure 4(f)). This data shows that disappearance of hepatic lipids seen in GW501516 treated groups between 4 and 8 weeks in non-tg animals (Figure 1(b)) follows accumulation of POPC. It might suggest that the build-up of critical levels of POPC required for PPAR*α* activation could be the cause of clearance of the liver TG, as a result of enhanced PPAR*α* activity in at least non-tg mice. However, further studies of POPC dynamics in both PPAR*α*-KO and PPAR*δ*-KO models are needed to confirm this hypothesis.

### 3.4. Early Hepatic Gene Expression Predicts Rate of Subsequent Weight Loss upon GW501516 Treatment

**Figure 5:**
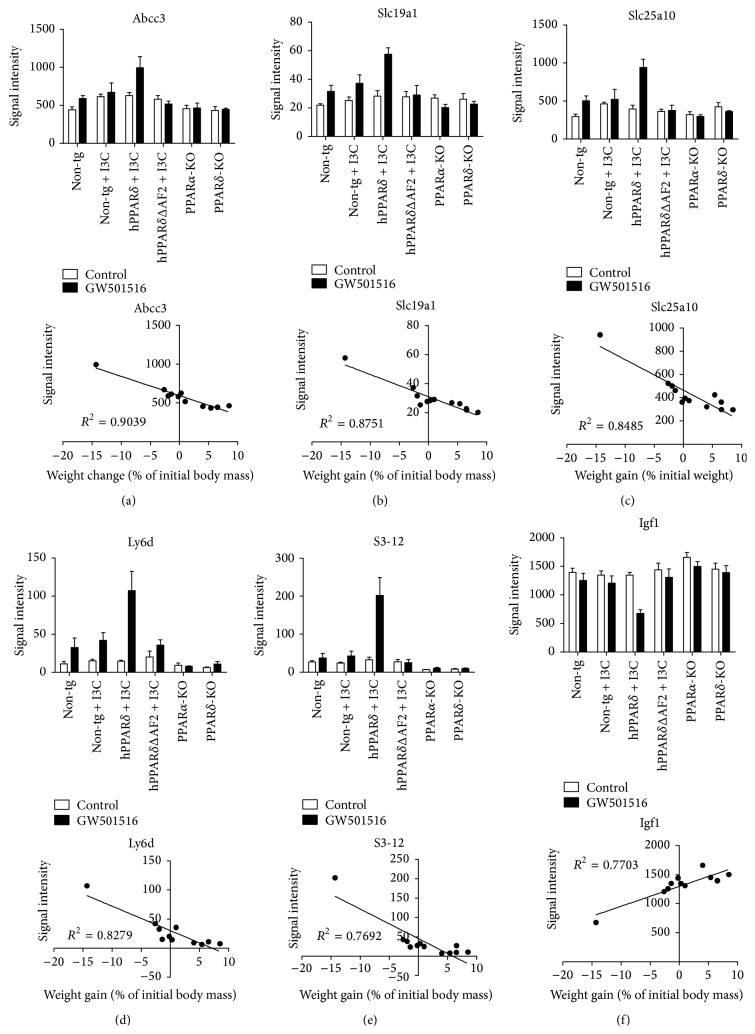
Genome-wide transcriptional profiling of GW501516 effects in various genetic models reveals strong correlation between liver gene expression after 5 days and successive weight gain rate after 2 weeks. Pattern of hepatic gene expression across the experimental genotypes in 5-day (bars graphs) and 2-week weight gain versus 5-day expression data for* Abcc3* (a),* Slc19a1* (b),* Slc25a10* (c),* Ly6d* (d),* S3-12* (e), and* Igf1* (f) (scatter graphs). Each point on the scatter graph represents mean value of each group (control or treated versus expression value) for given gene, *n* = 5 mice/group. Significance is indicated (^*∗*^ *P* ≤ 0.05; ^*∗∗*^ *P* ≤ 0.01; ^*∗∗∗*^ *P* ≤ 0.001; *t* test).

In nuclear receptor biology, initial activity of these transcription factors translate subsequently into metabolic and physiological changes. We assumed that short, 5-day study (no weight loss) would preserve nuclear-receptor-based transcriptional activity of whole sets of genes, not influenced yet by various physiological feedback loops and homeostatic mechanisms, which are likely to occur in long-term chronic ligand treatment coupled with substantial weight loss. The goal of this genome-wide transcriptional profiling was to demonstrate how early transcriptional actions in the liver involving PPAR*α*-PPAR*δ* tandem activities translate directly into phenotypic events in later stages. Six groups of mice were placed on specific diets: (1) non-tg on chow; (2) non-tg on chow + 0.0025% GW501516; (3) non-tg, expressing human PPAR*δ* (hPPAR*δ*) and expressing dominant negative derivative of PPAR*δ* (hPPAR*δ*ΔAF2) placed on chow + 0.25% I3C; (4) non-tg, hPPAR*δ*, and hPPAR*δ*ΔAF2 placed on chow + 0.25% I3C + 0.0025% GW501516; (5) PPAR*α*-KO and (6) PPAR*α*-KO mice placed on chow or chow + 0.0025% GW501516 (w/w). After 5 following days of experiment, mice were sacrificed, livers were harvested, and gene expression from this organ was analyzed utilizing microarrays. Twenty-seven genes were identified, whose pattern of expression was significantly correlated with rate of weight gain established from previous independent experiments (see Section 2.6); however, no genes were found (with FDR below 0.05), which would be significantly correlated with level of hepatic steatosis. The most numerous group of the genes significantly correlated with the weight loss turned out to be transmembrane transporters (Table 1). Abcc3 was one of the most significantly associated genes (Figure 5(a)). In study done by Hardwick et al. hepatic mRNA for Abcc3 was found to be elevated in human liver samples with confirmed Nonalcoholic Fatty Liver Disease (NAFLD) [33] and also has been reported in diabetic phenotype [34]. Other transporters found are* Slc19a1* (Figure 5(b)) which was reported also as a significant drug transporter, an important factor in response to methotrexate, a drug used for treatment of juvenile idiopathic arthritis [35], and* Slc25a10* (Figure 5(c)), the mitochondrial malate and succinate carrier.* Slc25a10* was previously shown to be essential for glucose stimulated insulin secretion (GSIS) [36]. Other examples involve genes involved in lymphocyte differentiation like* Ly6d *(Figure 5(d)), which has been previously associated with the degree of hepatic steatosis in mice [37]. Other genes, whose expression significantly correlated with weight loss, included the following: member of perilipin family* S3-12* (Figure 5(e)), a protein involved in coating intracellular lipid droplets (adipogenic marker), transcription regulating genes such as* Taf1d* (Table 1), or cell growth factors like insulin-like growth factor 1 (*Igf1*) (Figure 5(c)). It is consistent with study where low levels of* Igf1* were found in sera of patients with hepatic steatosis and this association was independent of alcohol consumption [38]. All, but* Igf1,* turned out to be negatively correlated with weight gain. Examples of pattern of expression and correlation graphs of chosen genes are shown in Figure 5.

## 4. Discussion

The ability of PPAR*δ* signalling to induce weight loss and improve plasma lipid profiles and glucose homeostasis was demonstrated previously [12, 16, 39]. PPAR*δ* is expressed ubiquitously [40], but in our animal model, the basal hPPAR*δ* transgene expression was very low in a wide range of tissues and highly inducible in the liver [21]. Consequently, the pronounced weight loss in hPPAR*δ* mice fed diet supplemented with GW501516 described by our group previously [21] and in this study might suggest that liver is important for the generation of these phenotypes.

### 4.1. PPAR*δ* Modulates Liver Lipid Metabolism by Direct and Indirect Way

Hitherto, the role of PPAR*δ* in liver steatosis remained an open question. For example, adenovirus-mediated overexpression of PPAR*δ* was enough to ameliorate hepatic steatosis in obese* db/db* mice in 7 days [18]. In another study,* db/db *mice treated with GW501516 for 14 days exhibited a 20% increase in liver TG [16]. In another work, authors also demonstrated increased TG content in livers of PPAR*δ* overexpressing mice [17]. Conversely, one of the recent works suggests that GW501516 treatment of mice fed HFD had no effect at all on liver TG [32]. Our study shows that hepatic steatosis was present in non-tg mice treated with PPAR*δ* agonist; however, it was strictly time-dependent and evident only after 4 weeks of treatment. In long term, the activation of PPAR*δ* by GW501516 turned out to be protective against liver steatosis.

The availability of PPAR*α* endogenous ligand (POPC) might be possible explanation for clearance of liver fat that was observed after long-term PPAR*δ* ligand treatment. Indeed, in our study, the levels of POPC were time-dependent in GW501516 treated non-tg mice livers (Figure 4(e)), suggesting the PPAR*δ* is “gating” the generation of POPC. Although POPC basal levels were significantly higher in PPAR*δ*-KO mice than in non-tg, this observation is still consistent with the findings of Adhikary et al., where they showed that, in the presence of ligand or genetic ablation of the receptor, a similar set of genes is upregulated [41]. Recent work stated that Fas is essential for synthesis of POPC [31]; however in our study, neither the gene expression data from RT-PCR (Figure 1(d)) nor microarray analysis (data not shown) demonstrated any evidence of Fas induction.

In hPPAR*δ* mice fed diet with GW501516, 2 weeks was sufficient for the accumulation of significant amount of lipids in the liver. A higher level of hepatic TG was also found in hPPAR*δ*ΔAF2 animals on normal chow diet. Recent findings [41, 42] suggest that repressor function is a major role for PPAR*δ*. Additionally, our findings show that hepatic fat level was reduced in hPPAR*δ*ΔAF2 mice treated with the PPAR*δ* agonist. This is consistent with previous observations that ligand binding to AF2 domain-deficient PPAR*δ* restores the repression function of this nuclear receptor, working in antagonist fashion, efficiently competing with endogenous mouse PPAR*δ* for PPRE binding sites.

Apart from direct accumulation from the diet [43], fatty liver appears as a result of increased glucose utilization feeding into* de novo* lipogenesis, with upregulation of Fas [44]. Jia et al. proposed this mechanism as an explanation for steatotic livers found in mice treated with GW501516, as an adaptive way to consume glucose. In our study, however, neither non-tg animals nor hPPAR*δ* mice with fatty livers had significantly elevated levels of hepatic Fas transcripts. Additionally, PPAR*γ*, a lipogenic marker [45], was downregulated in livers of the mice conditionally overexpressing hPPAR*δ*, treated with GW501516.

Mitochondrial *β*-oxidation is the prevailing oxidative pathway for the clearance of fatty acids [46]. Based on literature [47] and on Cpt1 and Acox1 gene expression results, there was no indication that *β*-oxidation processes in liver were interrupted by GW501516 treatment.

The remaining source of fatty acids in fatty liver is an influx from adipose tissue [48]. Fasting and exercise are characterized by amplified adipose tissue lipolysis and release of nonesterified fatty acids (NEFA) [49]. Excessive supply of NEFA, which are not oxidised, is reesterified by liver into TG and deposited in the cytoplasm of the hepatocyte [50, 51]. The PPAR*δ*-stimulated hepatic fatty accumulation we observed in our study was accompanied by substantial weight loss, if not preceded, with exceptions of PPAR*α*-KO and PPAR*δ*-KO mice fed GW501516, where neither weight loss nor liver steatosis was observed. The MRI scans confirmed that body weight reduction in hPPAR*δ* was due to fat mass decrease, rather than tempered appetite or lean mass reduction.

### 4.2. PPAR*α* and PPAR*δ* Role in Adipose Tissue Lipolysis

PPAR*δ* is known to be activated during prolonged fasting and increased physical activity [11]. PPAR*δ* agonists were even shown to work as an exercise mimetic substance and increase running endurance in adult mice [39]. However, pharmacological activation of PPAR*δ* forces adipose tissue to release fatty acids, which are not immediately required to power the body organs. Even during 36 hours of fasting in healthy human subjects and in mice exposed to 16 hours' fast temporary intrahepatic fat accumulation is observed [52, 53] and in patients with NAFLD, rapid weight loss deteriorates liver histopathology [54]. After intense exercise, mice also can accumulate significant level of TG in the livers, while the TG decrease in skeletal muscle [55]. Our study shows that pharmacological activation of PPAR*δ* leads to fasting or exercise-like weight loss and subsequent accumulation of TG in mouse liver.

This effect, however, requires PPAR*δ*-PPAR*α* collaboration. In a study where PPAR*α*-KO mice were fed with nonselective PPAR pan agonist, no weight loss was observed, but* ob/ob* mice treated with the same agonist lost approximately 20% of the body mass in 14 days [56]. Our work shows that although PPAR*α* ablation does not block upregulation of key PPAR*δ* target genes by GW501516 treatment, it abrogates some key PPAR*δ*-associated physiological effects. Specifically, based on the lack of weight loss in PPAR*α*-KO mice, we speculate that there is no apparent mobilization of lipid stores that is clearly happening in the non-tg mice treated with GW501516. Therefore it might suggest that functional PPAR*α* is essential for PPAR*δ*-driven mobilization of lipid stores from adipose tissue. However, the PPAR*δ* capability of raising HDL plasma levels [57] appeared to be not affected by PPAR*α* ablation and therefore seems to be PPAR*α*-independent.

Although Terada and colleagues [58] discussed two possibilities in which one of them was that GW501516 might act as direct activator of PPAR*α* in the absence of PPAR*δ*, weight loss or hepatic steatosis was not observed in GW501516 treated PPAR*δ*-KO mice (Figures 4(a) and 4(c)), demonstrating that activation of PPAR*α* by GW501516 is not sufficient to induce these responses. While GW501516 can act directly upon PPAR*α* to generate some phenotypes attributed also to PPAR*δ* such as lowering plasma TG or regulating common set of genes (such as Acox1), the collaboration between these two members of the same family is essential for weight loss initiation and hepatic lipid regulation (Figure 4(g)). The PPAR*δ*-assisted regulation of the PPAR*α* ligand POPC appears to play role in this process, at least in non-tg animals. However, this hypothesis needs to be confirmed in subsequent studies, as this current study lacks POPC dynamics data in PPAR*α*-KO and PPAR*δ*-KO mice upon GW501516 treatment.

Genome-wide transcriptional profiling demonstrates complexity of PPAR*α*-PPAR*δ* relations in regulating gene expression. For example, Plin2 and S3-12 have similar role in expressing proteins involved in lipid droplets coating. Whereas Plin2 expression was highly correlated with the level of hepatic lipids in non-tg, hPPAR*δ*, and hPPAR*δ*ΔAF2 mice, it was still highly inducible in PPAR*α*-KO livers where lipid accumulation was absent (Figures 3(e) and 3(f)). On the other hand, S3-12 expression was tempered efficiently by genetic KO of either receptor (Figure 5(e)) and correlated with the weight loss phenotype in these mice. S3-12 mRNA expression was previously shown to be 6-fold upregulated in fatty liver phenotype when PPAR*γ*1 was overexpressed in transgenic mice [59]. In our study, we also demonstrate that S3-12 mRNA expression is highly associated with PPAR*δ*-induced lipid fluctuations in mouse liver. Although after 5 days of treatment of mice with GW501516 some of the gene changes observed in this may be indirect, the main contract in the experiment was the gene ablation and therefore the time of activation is not important. Other genes (Table 1) found to be strongly correlated with predicted weight loss are all hepatocyte and possibly Kupffer cells expressed genes, which as our study shows have profound role in promoting weight loss. What is the exact link between PPAR*δ* and PPAR*α*, controlled gene expression in liver and weight loss, needs further study, considering the fact that it might suggest hormone-independent mechanism of regulation of rate of adipose lipolysis. This data would be consistent with previous studies that have suggested activation of PPAR*δ* promotes lipolysis via both: modulation of WAT adipose triglyceride lipase (ATGL) and enhanced hepatic production of ANGPTL4 [60, 61].

In summary, we show that liver specific expression of human PPAR*δ* in mouse liver promoted hepatic steatosis that was associated with significant loss of fat mass, suggesting extensive adipose tissue lipolysis and consequently an influx of fatty acids into the liver. This effect, however, is time-dependent and requires PPAR*α* signalling, with PPAR*α* working downstream of PPAR*δ*.

## Supplementary Material

Sequences of oligonucleotide primers and probes used in Taqman real time PCR.

## Figures and Tables

**Table 1:** List of the genes altered by GW501516 treatment in mouse liver.

Gene symbol	Entrez gene ID	Pearson *R*	*P* value	FDR	Gene description	Biological process
Abcc3	76408	−0.9649	3.97*E* − 07	0.003	ATP-binding cassette, subfamily C (CFTR/MRP)	Transmembrane transport
Slc19a1	20509	−0.9355	7.91*E* − 06	0.021	Solute carrier family 19 (sodium/hydrogen exchanger), member 1	Transmembrane transport
Slc25a10	27376	−0.9211	2.10*E* − 05	0.034	Solute carrier family 25 (mitochondrial carrier, dicarboxylate transporter)	Transmembrane transport
Abcc4	239273	−0.9096	4.07*E* − 05	0.033	ATP-binding cassette, subfamily C (CFTR/MRP)	Transmembrane transport
Slc16a5	217316	−0.9003	6.53*E* − 05	0.035	Solute carrier family 16 (monocarboxylic acid transporters)	Transmembrane transport
Srd5a3	57357	−0.9229	1.88*E* − 05	0.038	Steroid 5 *α*-reductase 3	Steroid catabolism
Cbr1	12408	−0.9055	5.04*E* − 05	0.034	Carbonyl reductase 1	Redox reactions
Grpel1	17713	−0.8953	8.31*E* − 05	0.035	GrpE-like 1, nuclear gene encoding mitochondrial protein	Protein anabolism
Ripk4	72388	−0.9023	5.94*E* − 05	0.034	Receptor-interacting serine-threonine kinase 4	Phosphorylation
Serhl	68607	−0.9201	2.24*E* − 05	0.03	Serine hydrolase-like (Serhl), mRNA.	Peroxisome function
Atxn10	54138	−0.8938	8.87*E* − 05	0.034	Ataxin 10	Nervous system development
Chchd6	66098	−0.8978	7.38*E* − 05	0.033	Coiled-coil-helix-coiled-coil-helix domain containing 6	Mitochondrial function
Ly6d	17068	−0.9099	4.02*E* − 05	0.036	Lymphocyte antigen 6 complex, locus D	Lymphocyte differentiation
Unc119	22248	−0.9030	5.75*E* − 05	0.035	Unc-119 homolog (*C. elegans*)	Lymphocyte differentiation
S3-12	57435	−0.8770	0.00018	0.048	Plasma membrane associated protein, S3-12	Lipid droplets coating
Gns	75612	−0.8952	8.33*E* − 05	0.033	Glucosamine (N-acetyl)-6-sulfatase	Glycosaminoglycan metabolic process
Gal3st1	53897	−0.8796	0.000163	0.05	Galactose-3-O-sulfotransferase 1	Glycolipid synthesis
Cpsf1	94230	−0.8935	8.99*E* − 05	0.033	Cleavage and polyadenylation specific factor 1	Gene expression
Taf1d	75316	−0.8913	9.93*E* − 05	0.033	TATA box binding protein (Tbp) associated factor	Gene expression
Sox12	20667	−0.8786	0.000169	0.05	SRY-box containing gene 12	Gene expression
S100a13	20196	−0.9524	1.78*E* − 06	0.007	S100 calcium binding protein A13	Cytokine secretion
Igf1	16000	0.8777	0.000175	0.048	Insulin-like growth factor 1	Cell growth
Nrg4	83961	−0.8991	6.95*E* − 05	0.033	Neuregulin 4	Cell growth
Pmm1	29858	−0.9147	3.08*E* − 05	0.035	Phosphomannomutase 1P	Carbohydrate metabolism
Prune	229589	−0.8866	0.000122	0.039	Prune homolog (*Drosophila*)	Carbohydrate metabolism
Tmem120a	215210	−0.9082	4.40*E* − 05	0.032	Transmembrane protein 120A	
C230029F24Rik	442837	−0.9141	3.19*E* − 05	0.032	PREDICTED: *Mus musculus* RIKEN cDNA C230029F24	
1600032L17Rik		−0.9001	6.62*E* − 05	0.033	PREDICTED: *Mus musculus* RIKEN cDNA 1600032L17	
2410012H22Rik	69747	−0.8784	0.00017	0.049	PREDICTED: *Mus musculus* RIKEN cDNA 2410012H22	

Genes were identified through correlation tests between microarray hepatic expression data from 5 days versus weight gain rate (% of initial body mass) after 2 weeks from several independent experiments.

FDR: false discovery rate.
