# Supplementary material for: PPARα Is Required for PPARδ Action in Regulation of Body Weight and Hepatic Steatosis in Mice
Source: PPAR Res. 2015 Oct 29;2015:927057. doi: 10.1155/2015/927057 (PMC4641930; doi:10.1155/2015/927057)
Supplement: Supplementary file 1 — Sequences of oligonucleotide primers and probes used in Taqman real time PCR. [file 927057.f1.pdf]

Supplementary Table 1 Sequences of oligonucleotide primers and probes used in Taqman real time PCR. All sequences are shown in 5'→3' order.

Gene	Forward primer	Taqman probe (FAM-TAMRA)	Reverse primer
human PPARδ	GGGACCACAGCATGCACTTC	CCAGCAGCTACACAGACCTCTCCCGG	TGCAGTTGGTCCAGCAGTGA
mouse ADRP	CAGCCAACGTCCGAGATTG	TGCCAGTGCCAGAGGTGCCGT	CACATCCTTCGCCCCAGT
mouse PPARα	GCGCAGCTCGTACAGGTCA	CAAGAAGACCGAGTCCGACGCAGC	TCTCTTGCAACAGTGGGTGC
mouse PPARγ	CTGACCCAATGGTTGCTGATTAC	AAATATGACCTGAAGCTCCAAGAATACCAAAGTG	TGGAGATGCAGGTTCTACTTTGA
mouse PPARδ	GACCAGAACACACGTTTCCTTC	AGCAGCTGTGCAGACCTCTCCCAGA	CCATCACAGCCCATCTGCA
mouse Acox1	TGACCGTTAAGGTCTTTGCAGA	AACTCCCCAAGATTCAAGACAGAGCCGT	AGGTTCCTCAGCACGGCTT
mouse CPT1	GGCTTAGTCGGGAGGCTCTG	AATCAACTCCTGGAAGAAACGCCTTATTCGAA	ACCCCTAAGGATGCCATTCTTG
mouse FAS	GGCATCATTGGGCACTCCTT	CCATCTGCATAGCCACAGGCAACCTC	GCTGCAAGCACAGCCTCTCT
mouse ANGPTL4	GCTTTGCATCCTGGGACGAG	ACTTGCTGGCTCACGGGCTGCTAC	CCCTGACAAGCGTTACCACAG
mouse PDK4	GGAAGTATCGACCCAAACTGTGA	CACTCAAAGGCATCTTGGACTACTGCTACCA	GGTCGCAGAGCATCTTTGC
